# Numerical Study of CH_4_ Generation and Transport in XLPE-Insulated Cables in Continuous Vulcanization

**DOI:** 10.3390/ma13132978

**Published:** 2020-07-03

**Authors:** Mohd Fuad Anwari Che Ruslan, Dong Joon Youn, Roshan Aarons, Yabin Sun, Shuyu Sun

**Affiliations:** 1 Computational Transport Phenomena Laboratory, King Abdullah University of Science and Technology, Thuwal 23955, Saudi Arabia; mohdfuadanwari.cheruslan@kaust.edu.sa; 2 Dow Chemical Europe, 8810 Horgen, Switzerland; raarons1@dow.com; 3 Dow Chemical (China) Investment Co., Ltd., Shanghai 200203, China; SYSun@dow.com

**Keywords:** cable insulation, XLPE, continuous vulcanization line, cross-linking reaction, byproduct degassing, reaction selectivity, heat transfer, CH_4_ diffusion

## Abstract

In this work, we apply a computational diffusion model based on Fick’s laws to study the generation and transport of methane (CH${}_{4}$) during the production of a cross-linked polyethylene (XLPE) insulated cable. The model takes into account the heating process in a curing tube where most of the cross-linking reaction occurs and the subsequent two-stage cooling process, with water and air as the cooling media. For the calculation of CH${}_{4}$ generation, the model considers the effect of temperature on the cross-linking reaction selectivity. The cross-linking reaction selectivity is a measure of the preference of cumyloxy to proceed either with a hydrogen abstraction reaction, which produces cumyl alcohol, or with a β-scission reaction, which produces acetophenone and CH${}_{4}$. The simulation results show that, during cable production, a significant amount of CH${}_{4}$ is generated in the XLPE layer, which diffuses out of the cable and into the conductor part of the cable. Therefore, the diffusion pattern becomes a non-uniform radial distribution of CH${}_{4}$ at the cable take-up point, which corresponds well with existing experimental data. Using the model, we perform a series of parametric studies to determine the effect of the cable production conditions, such as the curing temperature, line speed, and cooling water flow rate, on CH${}_{4}$ generation and transport during cable production. The results show that the curing temperature has the largest impact on the amount of CH${}_{4}$ generated and its distribution within the cable. We found that under similar curing and cooling conditions, varying the line speed induces a notable effect on the CH${}_{4}$ transport within the cable, while the cooling water flow rate had no significant impact.

## 1. Introduction

Cross-linked polyethylene (XLPE) is one of the most commonly used materials for power cable insulation because of its relatively low cost, simple processability, excellent electrical properties, and its resistance to chemicals and moisture [1]. XLPE is produced from polyethylene (PE) by a chemically activated cross-linking reaction, which improves PE’s thermal stability and mechanical properties at higher temperatures. PE tends to soften and melt at temperatures above 65 °C, making it unsuitable as an insulation material, especially for High Voltage (HV) and Extra-High Voltage (EHV) power cables [1,2].

**Figure 1:**
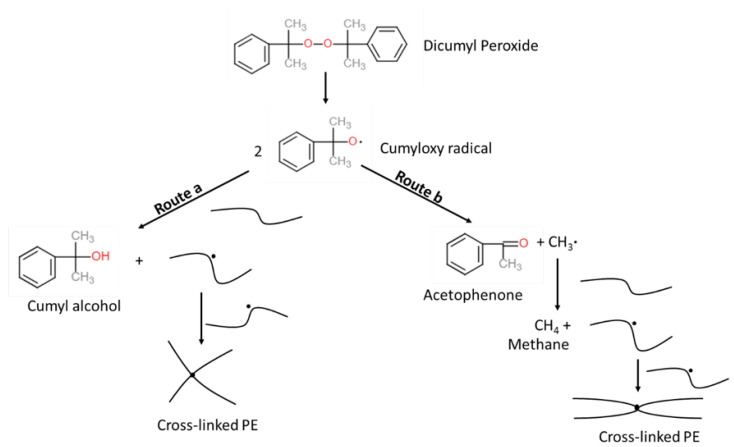
Dicumyl peroxide (DCP)-initiated polyethylene (PE) cross-linking reaction scheme compiled from Andrews et al. [1] and Sun et al. [13].

In the cable industry, this cross-linking reaction typically uses peroxide as a reaction initiator. Dicumyl peroxide (DCP) is one of the preferred initiators due to its relatively fast decomposition rate at normal cable processing temperatures [3]. Figure 1 illustrates the PE cross-linking reaction scheme using DCP as an initiator. While improving the thermal and mechanical properties of PE, the cross-linking reaction also produces several unwanted byproducts. These byproducts may have detrimental effects on the cable’s mechanical and electrical performance, and also raise safety issues because of the flammability of the byproducts [1,4,5,6]. To reduce the concentration of byproducts in the cable to an acceptable level, the cable must undergo additional thermal treatment processes after production, which is known as byproduct degassing. In the degassing process, the cable is placed in a degassing chamber and the chamber temperature is typically controlled to around 70 °C to facilitate byproduct removal from the power cable. This process is expensive and time consuming, and is considered to be a critical bottleneck for cable production, especially for HV and EHV power cables, which generally have thicker insulation [3].

Several studies have been performed, either experimentally [7,8,9,10,11,12] or numerically [1,3,12,13,14,15,16,17] to better understand byproduct transport and removal during the degassing process. The initial distribution of a byproduct in a cable, which depends on byproduct generation and transport during cable production, has been shown to affect the degassing process [3]. Several studies have simulated cable production conditions [18,19,20,21]; however, these studies have focused only on simulating the temperature and cross-linking completion during cable degassing. The transport of byproducts generated during the cross-linking reaction after the cable extraction has not yet been closely examined in terms of the detailed production conditions. Understanding the generation and transport of byproducts under varying cable production conditions could provide essential information for the design of optimal cable production and degassing processes.

The objective of this study is to apply a computational diffusion model with various *in-situ* cable manufacturing conditions and study the transport of the byproducts that are generated by the PE cross-linking reaction during cable production, before the cables are degassed. We focus only on the byproduct methane (CH${}_{4}$) and its transport, primarily because of methane’s explosive combustion risk. However, this model can be easily applied using different diffusion-related parameters to consider the transport of other byproducts such as acetophenone (AP) and cumylalcohol (CA). Using this model, we perform several parametric studies to numerically determine and characterize the critical cable production parameters that affect CH${}_{4}$ generation and transport processes during cable extraction and production.

## 2. Cable Production Condition

**Figure 2:**
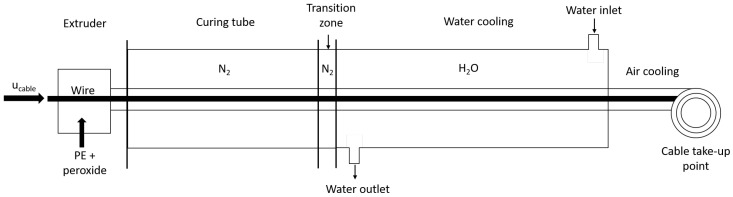
Schematic of power cable production line.

Figure 2 shows a schematic of a general cable production line, namely a continuous vulcanization (CV) line. During the cable production process, the conductor entering into the extruder undergoes a triple extrusion process, in which the inner semiconductor layer, the PE insulation layer, and the outer semiconductor layer are applied simultaneously over the conductor. The simultaneous extrusion is performed to construct a smooth interface between the insulation and the conductors, thus avoiding concentrated electrical stress and minimizing contamination during extrusion [2].

The extruded cable moves into the heated, high-pressure tube filled with nitrogen, known as the curing tube, where the cross-linking reaction takes place. The high temperature in the curing tube facilitates the decomposition of the peroxide initiator. In the curing tube, the heat is transferred to the cable through radiation from the tube wall and the nitrogen gas. Shugai [19] showed that radiation is the primary heat transfer mechanism in the curing tube; the maximum influence of heat transfer through nitrogen on the cable temperature is within 5%. The curing tube is divided into several heating zones, where each heating zone temperature is adjusted individually for optimal production. At the end of the curing tube, there is a short segment, referred to as the transition zone, which is filled with high-pressure nitrogen but not heated.

The heated cable is cooled to ambient temperature in two cooling stages. In the first cooling stage, the cable is cooled with water, typically flowing in the opposite direction of the cable production line. In this stage, the cable temperature decreases rapidly through forced convection heat transfer. The pressure in this section is typically similar to the curing tube pressure. In the second cooling stage, the cable is exposed to ambient temperature, where free convection heat transfer occurs from the cable to the ambient air. This cooling process continues until the cable take-up point where the cable is securely bound on a reel and ready for degassing.

## 3. Mathematical Model

To begin, we make several assumptions to effectively account for the cable configurations and CV line production conditions. The following are the main assumptions applied to construct this model:

- The cable layers are homogeneous and isotropic in terms of heat transfer and byproduct diffusion.
- The DCP is uniformly distributed across the insulation layer.
- The heat generated from the cross-linking reaction, 900 kJ·kg${}^{-1}$ of DCP is negligible compared to the amount of energy supplied by the curing tube [19,20].
- The cable layers are stuck tightly together, and there is no free space between the layers to allow for diffusion of the byproduct.
- The byproduct diffusion follows Fick’s laws, where the diffusion coefficient depends only on the temperature.
- The byproduct concentration at the cable surface is set to zero in both the curing tube and cooling sections because the byproducts released from the cable are removed by the surrounding fluid circulations.

Due to the symmetric geometry of the cable and CV line, we built the computational model applying a finite element-based approximation with a two-dimensional axisymmetric condition. Therefore, the volume of the cable and CV production line are defined as two-dimensional planes while the rotating axis are positioned at the center of the cable.

### 3.1. Cross-Linking Reaction

For a steady-state condition, the DCP transport can be computed using Equation (1).

(1) $$u\cdot \nabla c_{dcp}=r_{p}$$

where *u* is the cable production line speed, $c_{dcp}$ is the DCP concentration, and $r_{p}$ is the peroxide decomposition rate. In this study, $r_{p}$ is modelled as a first order kinetic as shown in Equation (2).

(2) $$r_{p}=-A_{p}\exp\left(\frac{-E_{a,p}}{RT}\right)c_{dcp}$$

where $A_{p}$ and $E_{a,p}$ are the Arrhenius pre-exponential factor and activation energy, respectively. Typically, $r_{p}$ is defined in terms of the peroxide conversion, more commonly referred to as the degree of cross-linking, α, as in Equation (3).

(3) $$\alpha =1-\frac{c_{dcp}}{c_{dcp,i}}$$

where $c_{dcp,i}$ is the peroxide concentration at the CV line inlet. Using this definition, the DCP transport equation and cross-linking reaction rate defined in Equations (1) and (2) can be rewritten in terms of the degree of cross-linking, as in Equations (4) and (5).

(4) $$-u\cdot \nabla \left(\alpha c_{dcp,i}\right)=r_{p}$$

(5) $$r_{p}=-A_{p}\exp\left(\frac{-E_{a,p}}{RT}\right)\left(1-\alpha \right)c_{dcp,i}$$

As demonstrated in Figure 1, the cumyloxy radical generated from DCP decomposition can either attack the PE chain by abstracting a hydrogen atom from the PE molecule to produce CA and a PE radical (route *a*), or rearrange itself (β-scission) to produce AP and a methyl radical (route *b*). To determine the amount of each byproduct generated, the selectivity of route *a* and route *b* reactions must be known. In this study, the route *b* reaction selectivity, $S_{b}$, is defined as the ratio of route *b* reaction rate over the sum of route *a* and route *b* reaction rates, as in Equation (6).

(6) $$S_{b}=\frac{r_{b}}{r_{a}+r_{b}}$$

where $r_{a}$ and $r_{b}$ are the reaction rate for route *a* and route *b*, respectively. The route *a* and route *b* reaction rates are defined in Equations (7) and (8), respectively.

(7) $$r_{a}=A_{a}\exp\left(\frac{-E_{a,a}}{RT}\right)c_{ph}\left(2\alpha c_{dcp,i}\right)$$

(8) $$r_{b}=2A_{b}\exp\left(\frac{-E_{a,b}}{RT}\right)\left(2\alpha c_{dcp,i}\right)$$

where $A_{a}$ and $A_{b}$ are the respective pre-exponential factors for the route *a* and route *b* reactions, $E_{a,a}$ and $E_{a,b}$ are the respective activation energies for the route *a* and *b* reactions, and $c_{ph}$ is the ethylene monomer concentration. Using the reaction rate definitions in Equations (7) and (8), Equation (6) can be rewritten as in Equation (9).

(9) $$S_{b}=\frac{A_{b}\exp\left(\frac{-E_{a,b}}{RT}\right)}{A_{a}\exp\left(\frac{-E_{a,a}}{RT}\right)c_{ph}+A_{b}\exp\left(\frac{-E_{a,b}}{RT}\right)}$$

### 3.2. Heat Transfer

Generally, we solve the energy balance equation as presented in Equation (10) to estimate the temperature profile in the entire cable domain regardless of the production phase. Since the cable continuously moves along the production line, the production line speed, *u*, is combined with the thermo-physical properties in the term on the left, while we perform the analysis as a steady state condition. The amount of heat generated from the cross-linking reaction is ignored due to its negligible effect compared to the other heat sources [19,20].

(10) $$\rho C_{p}u\cdot \nabla T=\nabla \cdot \left(k\nabla T\right)$$

where ρ is the density, $C_{p}$ is the specific heat capacity, and *k* is the thermal conductivity. The corresponding thermo-physical properties are applied to solve the equation for each cable component. In the following subsection, we explain the additional boundary conditions for each CV line segment.

#### 3.2.1. Curing Tube Conditions

The nitrogen temperature distribution in the curing tube and transition zone is estimated using the energy balance equation presented in Equation (10). In the model, we assume that nitrogen is in a relatively static phase with minor buoyancy, (i.e., $u_{N2}=0$), hence the equation can be simplified by removing the convection term associated with the nitrogen speed in computing the nitrogen temperature profile.

The curing tube wall at higher temperature acts as the most significant heat source that increases the cable temperature. Therefore, the radiation heat transfer rate, $q_{rad}$, is computed using Equation (11) and assigned along the cable surface.

(11) $$q_{rad}=\frac{\sigma (T_{t}^{4}-T_{c}^{4})}{\frac{1}{\epsilon_{c}}+\left(\frac{d_{c}}{d_{t}}\right)\left(\frac{1}{\epsilon_{t}}-1\right)}$$

where σ is the Stefan–Boltzmann constant, $T_{t}$ is the tube wall temperature, $T_{c}$ is the cable temperature, $d_{c}$ is the cable diameter, $d_{t}$ is the tube diameter, $\epsilon_{c}$ is the cable surface emissivity, and $\epsilon_{t}$ is the tube emissivity.

The values of 0.7 and 1.0 are used for $\epsilon_{c}$ and $\epsilon_{t}$, respectively, according to Shugai and Yakubenko [20]. The curing tube wall temperature, $T_{cw}$ is assigned to $T_{t}$ in Equation (11), and the $q_{rad}$ is computed based on $T_{t}$ and the iterated $T_{c}$ values. Note that $T_{cw}$ is also used to compute the nitrogen temperature profile in the curing tube.

Lastly, we assume that the cable temperature at the curing tube inlet increases to a certain level due to a conductor pre-heater and insulation applying temperature as in Equation (12).

(12) $$T_{c}^{inlet}=T_{ph}$$

where $T_{ph}$ is the conductor pre-heater temperature and the superscript inlet indicates the location of the curing tube inlet.

#### 3.2.2. Transition Zone Conditions

Although the transition zone is directly exposed to ambient air, the temperature within the transition zone is normally considerably higher than the ambient temperature due to the high cable temperature exiting the curing tube and the conduction heat transfer between the curing tube wall and transition zone wall. The casing wall temperature in the transition zone and the water cooling section needs to be computed in order to determine the rate of heat dissipation to the ambient within these sections. According to the heat transfer mechanism, the casing wall temperature in the transition zone is computed using Equation (10) with a boundary condition defined as in Equation (13) at the transition zone inlet.

(13) $$T_{tw}^{inlet}=T_{cw}^{outlet}$$

where $T_{tw}^{inlet}$ is the wall temperature at the transition zone inlet and $T_{cw}^{outlet}$ is the wall temperature of the curing tube at the outlet. Since the casing wall is static (i.e., $u_{tw}=0$), the convection term is removed in Equation (10) to compute the casing wall temperature profile.

As $T_{tw}^{inlet}$ is always much greater than the ambient temperature, $T_{amb}$, we expect to see significant thermal energy dissipation from the casing wall to the ambient air through radiation and natural convection. We calculated the radiation heat transfer rate from the casing wall to the ambient air, $q_{rad}^{amb}$, using Equation (14) according to Holman [22].

(14) $$q_{rad}^{amb}=\sigma \epsilon_{t}\left(T_{tw}^{4}-T_{amb}^{4}\right)$$

Moreover, the convective heat transfer rate between the casing wall and ambient, $q_{conv}^{amb}$, is computed as in Equation (15).

(15) $$q_{conv}^{amb}=h\left(T_{tw}-T_{amb}\right)$$

where *h* is the convective heat transfer coefficient. In this study, we considered the CV line to be vertically oriented so that Equation (16) could be used to estimate *h* for the natural convection heat transfer at this interface [22].

(16) $$Nu=\frac{hd}{k}=0.59{\left(GrPr\right)}^{0.25}$$

where Gr and Pr are the Grashof and Prandtl number, respectively. The Grashof and Prandtl numbers are computed using Equations (17) and (18), respectively.

(17) $$Gr=\frac{g\varepsilon \Delta TL^{3}}{\nu^{2}}$$

(18) $$Pr=\frac{C_{p}\mu }{k}$$

where *g* is the gravitational acceleration, ε is the coefficient of thermal expansion, ΔT is the difference between the surface and bulk temperature, *L* is the characteristic length, ν is the kinematic viscosity and μ is the dynamic viscosity.

Similar to the curing tube section, we expect to see significant radiation heat transfer between the cable surface and casing wall. The radiation heat transfer rate supplied from the casing wall to the cable surface is computed using Equation (11) where $T_{tw}$ is used as $T_{t}$. This radiation heat transfer rate is directly assigned along the cable surface. Unlike the curing tube section in which the wall temperature is directly controlled as a modelling condition, this radiation heat removal from the casing wall is assigned along the casing wall and is included in the casing wall temperature computation.

#### 3.2.3. Cooling Section Conditions

In the water cooling section, the heat is removed from the cable through forced convection. The convective heat flux from the cable surface to water, $q_{conv}^{w}$, is computed using Equation (19). Note that the average water temperature, $T_{w}^{ave}$, instead of water temperature at the interface is used in this computation.

(19) $$q_{conv}^{w}=h\left(T_{c}-T_{w}^{ave}\right)$$

The heat transfer coefficient, *h*, for the forced convection heat transfer at this interface is computed using the correlation defined as in Equation (20) [18,22].

(20) $$Nu=\frac{hd}{k}=0.023Re^{0.8}Pr^{n}$$

where Re is the Reynolds number. The Reynolds number is computed using Equation (21).

(21) $$Re=\frac{\rho ud}{\mu }$$

The coefficient, *n*, in Equation (20) is 0.3 for cooling of the fluid and 0.4 for heating of the fluid.

Similarly, the convective heat flux along the interface between the casing wall and water is computed using Equation (19) with the heat transfer coefficient, *h*, for the forced convection heat transfer at this interface computed using Equation (20). As the casing wall is exposed to ambient temperature, additionally, heat transfer occurs between the casing wall and ambient air through natural convection too. The convective heat flux at this interface is computed using Equation (15) while the heat transfer coefficient, *h*, for the natural convection heat transfer at this interface is computed using Equation (16). With all the described conditions, the temperature profile of the cable, water and casing wall are estimated using Equation (10).

For the air-cooling section, the natural convection heat transfer between the cable and ambient air at the cable surface becomes the only factor that affects the cable temperature, which is calculated in the same way as the water-cooling section using Equations (15) and (16).

### 3.3. Byproduct Transport

As mentioned earlier, here, we estimated CH${}_{4}$ transport only in terms of the issues of safety. The transport mechanism for CH${}_{4}$ is diffusion, which can be computed using Equation (22).

(22) $$\frac{\partial c_{m}}{\partial t}+\nabla \cdot \left(-D\nabla c_{m}\right)+u\cdot \nabla c_{m}=r_{m}$$

where *D* is the diffusion coefficient, $c_{m}$ is the CH${}_{4}$ concentration, and $r_{m}$ is the rate of CH${}_{4}$ generation. As shown in Equation (23), $r_{m}$ depends on the route *b* reaction selectivity, $S_{b}$, which is defined in Equation (9), and the cross-linking reaction rate, $r_{p}$, which is defined in Equation (5).

(23) $$r_{m}=2S_{b}r_{p}c_{dcp,i}$$

The diffusion coefficient, *D*, is defined as the Arrhenius expression, as shown in Equation (24).

(24) $$D=A_{d}\exp\left(-\frac{E_{a,d}}{RT}\right)$$

where $A_{d}$ and $E_{a,d}$ are the pre-exponential factor and activation energy for the diffusion coefficient, respectively. In this study, we considered a steady-state cable production process. Hence, Equation (22) can be simplified to Equation (25).

(25) $$\nabla \cdot \left(-D\nabla c_{m}\right)+u\cdot \nabla c_{m}=r_{m}$$

In addition, the zero-CH${}_{4}$ concentration is assigned at the CV line inlet and along the cable surface as the boundary condition.

(26) $$c_{m}=0$$

## 4. Simulation Parameters

### 4.1. Cable and CV Line Specifications

**Figure 3:**
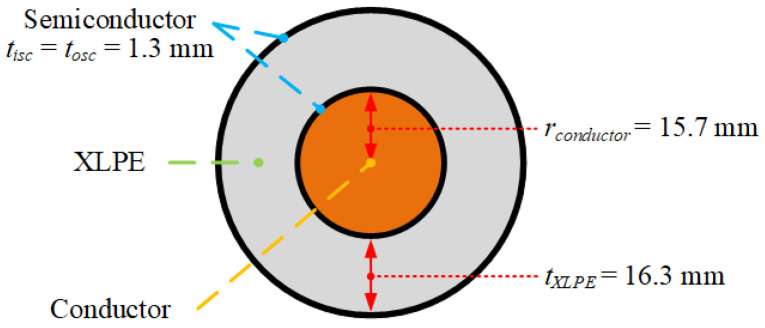
The 132 kV cable specification.

To perform representative degassing analyses, we first chose a single core HV cable with a typical XLPE insulation configuration and a 132 kV power cable with a 16.3 mm XLPE insulation layer, as demonstrated in Figure 3. The curing tube, transition zone, and water-cooling section diameters and the casing wall thickness were set to 200 and 10 mm, respectively. Additionally, Table 1 shows the dimensions for each of the CV line sections according to Kosar and Gomzi [18].

Table 2 lists the CV line operating conditions considered in this study. In this study, the ambient temperature, $T_{amb}$, was set to 25 °C.

### 4.2. Cross-Linking Reaction

In this study, we used the values of $9.24\times 10^{15}$ s${}^{-1}$ and 152.67 kJ·mol${}^{-1}$ as the Arrhenius parameters for the cross-linking reaction, $A_{p}$ and $E_{a,p}$, respectively [23]. The Arrhenius parameters for both reaction routes shown in Figure 1 were required for reaction selectivity computation as per Equation (9). As the β-scission reaction for non-polar solvents is mainly a function of temperature [24], we used the cumyloxy β-scission reaction parameters computed for reactions in chlorobenzene and cumene [25] as an estimate for the cumyloxy β-scission reaction in PE. These β-scission reaction parameters were $2.29\times 10^{12}$ s${}^{-1}$ and 35.9 kJ·mol${}^{-1}$ for *A* and $E_{a}$, respectively [25]. The kinetic parameters for the hydrogen abstraction reaction was estimated based on the kinetic parameters for β-scission reaction and the byproduct ratio data for DCP decomposition in PE using Equation (27).

(27) $$\ln\frac{c_{ca}}{c_{ap}c_{ph}}=\ln\frac{A_{a}}{A_{b}}-\frac{E_{a,a}-E_{a,b}}{RT}$$

where $c_{ca}$ and $c_{ap}$ are the CA and AP concentrations, respectively.

**Figure 4:**
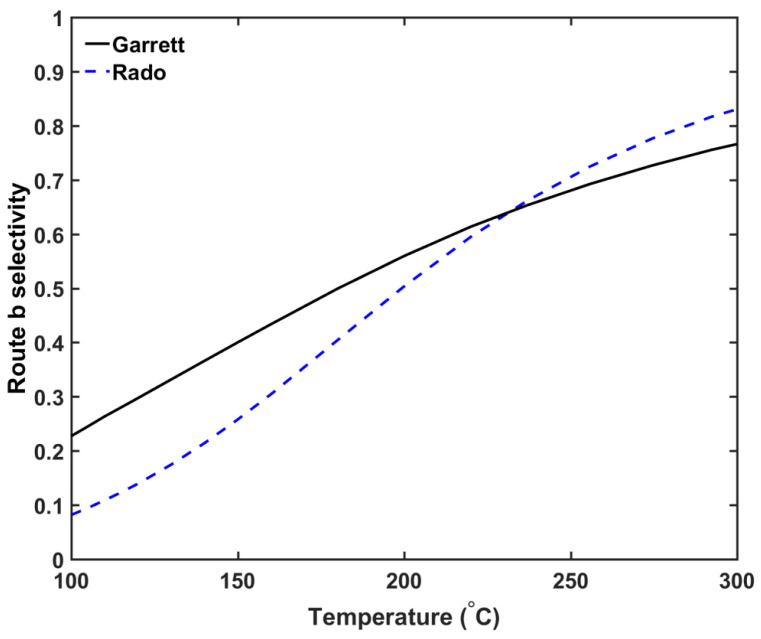
Route *b* reaction selectivity profile [28,29].

The kinetic parameters for the hydrogen abstraction reaction computed using this approach are shown in Table 3. Note that there is a significant difference between the computed reaction kinetic parameters from the two sets of experimental data. One possible explanation for this difference is the composition difference between the PE samples used in these experiments, where different C–H bonds may cause different kinetic parameters in the hydrogen abstraction reaction. Both sets of hydrogen abstraction kinetic parameters show similar trends whereby the route *b* selectivity increases as the curing temperature increases, as demonstrated in Figure 4. This trend is consistent with DCP decomposition data in other solvents [26,27].

### 4.3. Thermophysical Properties

The thermal and mechanical properties used for each cable components in this study are listed in Table 4.

### 4.4. Methane Diffusion

**Figure 5:**
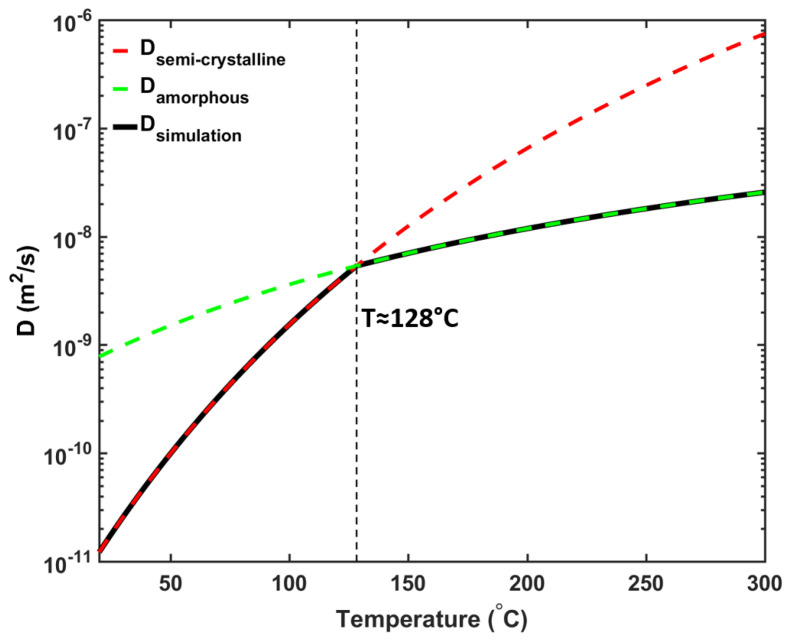
CH${}_{4}$ diffusion coefficient variations in XLPE as semi-crystalline [13] (red line) and amorphous [35] (green line) phases of the XLPE, which were then combined to represent the diffusion coefficient variation applied in the simulation (black line).

During cable production, the temperature at the cable surface can increase up to the pre-defined curing tube temperature, i.e., 300 °C, in the curing tube, followed by a reduction to ambient temperature at the cable take-up point. Due to the significant temperature changes, the XLPE must undergo a phase transition during cable production. Normally, the XLPE has a semi-crystalline structure, but the crystalline structure starts to melt at around 70 °C [1]. Since the crystalline structure may significantly affect the overall diffusion property in the XLPE [30,31,32], knowing the effect of phase changes on the diffusion coefficient is important for the accurate analysis of byproduct generation and transport. In this study, hence, we combined two available diffusion coefficient profiles that represent the experimentally measured semi-crystalline structures [17] and the amorphous phases, as estimated by molecular dynamic simulations [33,34,35] to cover the different temperature ranges. Figure 5 shows the diffusion profile obtained using this approach. The intersection point of the two diffusion profiles occurred at approximately 128 °C, thus defining the transition temperature between the two different XLPE phases in this work.

Beside temperature and crystalline phase, the diffusion coefficient can also be influenced by other variables such as cross-linking density and CH${}_{4}$ concentration. One possible approach to characterize these effects is based on the free volume theory [36,37,38]. However, we chose not to use this method mainly due to lack of available data to characterize CH${}_{4}$ diffusion based on the cable manufacturing condition used in this study. Also, the simulation results from [35] show that only a small amount of cross-linking density is observed for these cable manufacturing conditions (approximately 4.2% for system with 2.5% peroxide content), and under these conditions, cross-linking density has a minor effect on the CH${}_{4}$ diffusion. Lastly, the molecular dynamic simulation results from Dutta and Bhatia [39] show that CH${}_{4}$ concentration has a negligible effect on CH${}_{4}$ diffusion in PE.

In addition, Youn et al. [40] conducted an inverse calculation using a set of experimental CH${}_{4}$ concentration data to find the diffusion characteristics of the conductor, and found that the conductor also plays an important role in the overall degassing process due to its relatively large diffusion coefficient (which was only 41.56% of XLPE’s diffusion coefficient). Therefore, we also added the cable conductor that is fully connected to the surrounding polymer layers for the entire heat transfer and CH${}_{4}$ diffusion simulations, and we applied the diffusion coefficient of the cable conductor presented in Youn et al. [40] to the CH${}_{4}$ transport analysis in the paper.

## 5. Results and Discussion

### 5.1. Temperature and Cross-Linking Completion Profile

**Figure 6:**
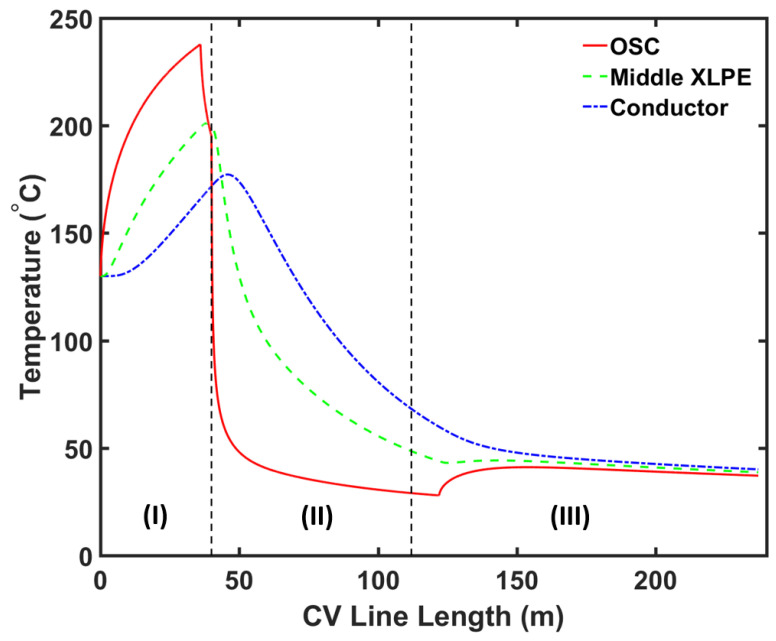
CV line temperature profile at different distances from the cable centre. Segment (I), (II) and (III) correspond to the curing tube and transition zone, water-cooling, and air-cooling segments, respectively.

**Figure 7:**
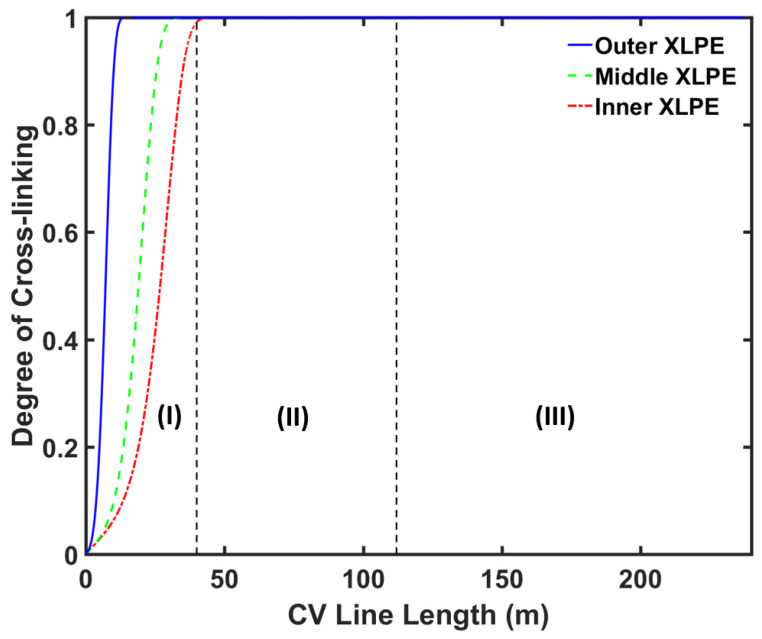
CV line cross-linking profile at different distances from the cable centre. Segments (I), (II) and (III) correspond to the curing tube and transition zone, water-cooling, and air-cooling segments, respectively.

**Figure 8:**
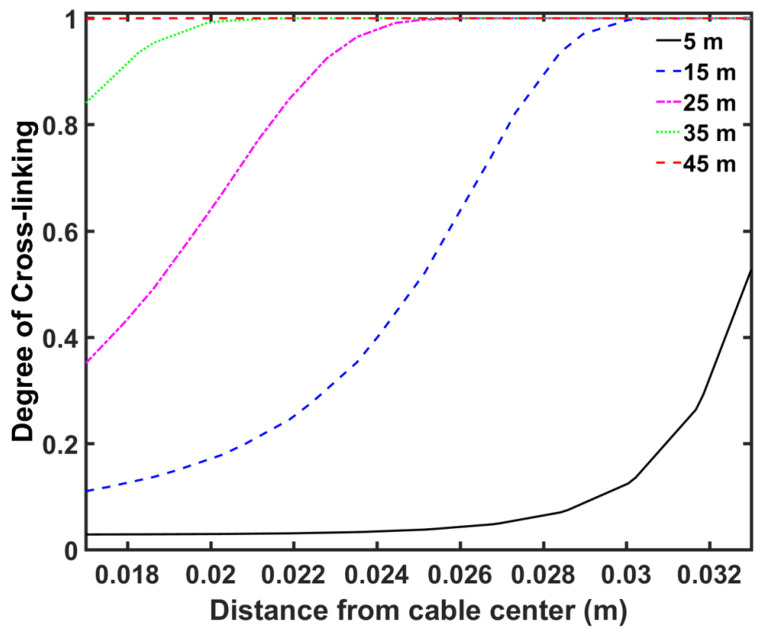
Radial cross-linking profile at different distances from the CV line inlet.

Figure 6 and Figure 7 show the axial temperature and cross-linking completion profiles at different locations within the cable, while Figure 8 shows the radial cross-linking completion profile at different locations across the CV line. The temperature at the cable surface increased rapidly in the curing tube section and reached up to approximately 240 °C at the exit of the curing tube. The high cable temperature facilitated the cross-linking reaction in the insulation layer. The heat received at the cable surface was transferred internally through conduction. As the XLPE and SC had low thermal conductivity, the temperature at the inner cable layer increased at a slower rate compared to the temperature increase at the cable surface. This led to a slower cross-linking rate in the inner side of the insulation compared to the rate in the outer side. The cross-linking reaction occurred mainly in the curing tube section, where we observed cross-linking completion above 90% throughout the insulation layer at the curing tube exit.

The temperature at the cable surface began to drop in the transition zone as some heat dissipated externally to the surrounding nitrogen and the casing wall. However, the temperature at the inner cable layer still increased in the transition zone because the temperature at the cable surface was still greater than the temperature at the inner side of the cable. An additional cross-linking reaction was completed in this section too, causing the cross-linking completion to reach above 99% throughout the insulation layer at the transition zone exit.

The cable temperature decreased more rapidly as the cable entered the water-cooling section. Again, we observed a slower rate of temperature change at the inner side of the cable due to the low thermal conductivity of XLPE and SC. At the water-cooling section’s exit, the cable surface temperature was around 28 °C while the conductor temperature was around 60 °C.

At the air-cooling section, the temperature at the cable surface initially increased because of the amount of heat supplied from the inner side of the cable, which was greater than the amount of heat removed by free convection to ambient air. The temperature across the cable gradually approached an equilibrium condition in this section. At the cable take-up point, the cable surface temperature was approximately 37 °C while the conductor temperature was approximately 40 °C. We observed cross-linking completion above 99% throughout the insulation layer at the cable take-up point.

### 5.2. Methane (CH_4_) Generation and Transport

**Figure 9:**
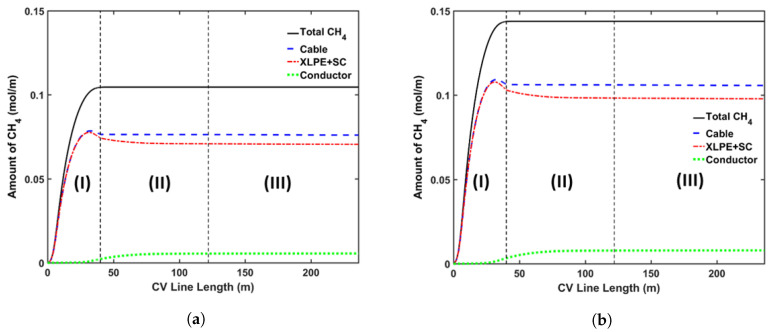
Axial CH_4_ concentration profile across the CV line, computed based on two different cross-linking reaction selectivity profiles. The reaction selectivity profile based on Rado’s data [28] and Garrett’s data [29] was used for computing the reaction selectivity in (**a**,**b**), respectively. Segments (I), (II), and (III) correspond to the curing tube and transition zone, water-cooling, and air-cooling segments, respectively.

**Figure 10:**
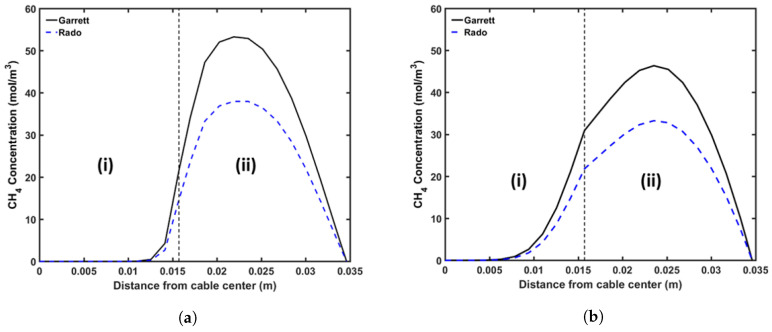
Radial CH_4_ concentration profile at (**a**) curing tube exit, (**b**) cable take-up point. segment (i) corresponds to conductor layer while segment (ii) corresponds to XLPE and SC layers.

In this study, the DCP concentration in the insulation layer was set to 2 wt%. Figure 9 shows the axial CH${}_{4}$ concentration profile and Figure 10 shows the radial CH${}_{4}$ profile at the curing tube exit and cable take-up point. We considered both reactions’ selectivity profiles, as shown in Figure 4, to estimate the CH${}_{4}$ concentration in this study. Table 5 lists the amount of CH${}_{4}$ presence in the cable at the cable take-up point.

Our results showed that the CH${}_{4}$ generation in the cable was significantly influenced by the reaction selectivity. Rado’s reaction selectivity model [28] produced around 72.7% of the CH${}_{4}$ produced in Garret’s model [29], as shown in Table 5. As mentioned previously, the PE sample composition may be one of the possible reasons for the different reaction selectivity observed from the experiments [28,29]. Hence, it is important to properly quantify and determine the cross-linking reaction selectivity for the PE composition used for cable production. Although the CH${}_{4}$ concentration in the cable was different, the proportional amounts of CH${}_{4}$ stored in each cable component compared to the total CH${}_{4}$ generated in the entire cable (%CH${}_{4}$) were relatively similar for both selectivity profiles. This was expected since the CH${}_{4}$ distribution within a cable mainly depends on the CH${}_{4}$ diffusion process, which is defined by the cable temperature and not by the amount of CH${}_{4}$ generated.

The axial CH${}_{4}$ concentration profile shows that a significant amount of CH${}_{4}$ was removed from the cable in the curing tube and the transition zone sections. The high cable temperature facilitated this removal as the CH${}_{4}$ diffusion coefficient increased exponentially with temperature. The amount of CH${}_{4}$ released from the cable was negligible in the cooling section. Overall, approximately 26.9% of the CH${}_{4}$ generated from the cross-linking reaction was removed from the cable during cable production.

The simulations also show that a significant amount of CH${}_{4}$ diffused into the conductor. This diffusion process mainly occurred in the water-cooling section because the greater temperature in the conductor compared to the temperature nearer the cable surface expedited the diffusion process from the insulation layer into the conductor. In the air-cooling section, we observed very minor diffusion between the conductor and the insulation as the cable temperature decreased, and the concentration gradient between the domains became negligible. Approximately 5.4% of the CH${}_{4}$ generated from the cross-linking reaction diffused into the conductor during cable production.

The non-uniform radial concentration distribution found at the cable take-up point presented a very similar pattern to the byproduct concentration profiles obtained from the experiments [8].

### 5.3. Parametric Study

We performed a series of parametric studies to analyze the effect of the CV line operating conditions on the byproduct transport process during cable production. We applied different curing temperatures, line speeds, and cooling water flow rates to the actual purpose of each cable production phase. For the base case definition, we applied the reaction selectivity computed from Garrett’s experiment data, ref. [29] and the CV line operating conditions, as listed in Table 2.

#### 5.3.1. Curing Temperature

**Figure 11:**
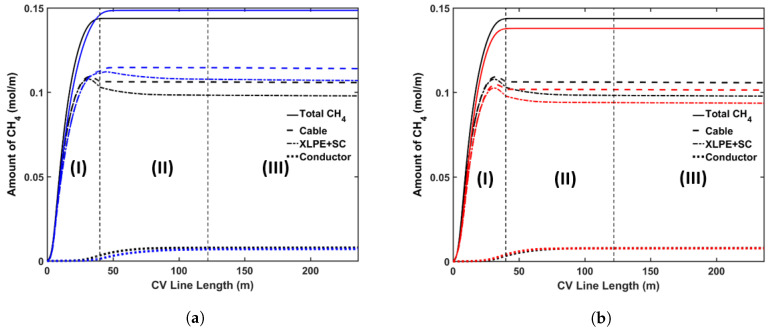
Axial CH_4_ concentration profile across CV line for curing temperature study. The black line represents base case, blue line in (**a**) represents Case 1a, and red line in (**b**) represents Case 1b. Segment (I), (II) and (III) correspond to curing tube and transition zone, water cooling, and air cooling segments, respectively.

**Figure 12:**
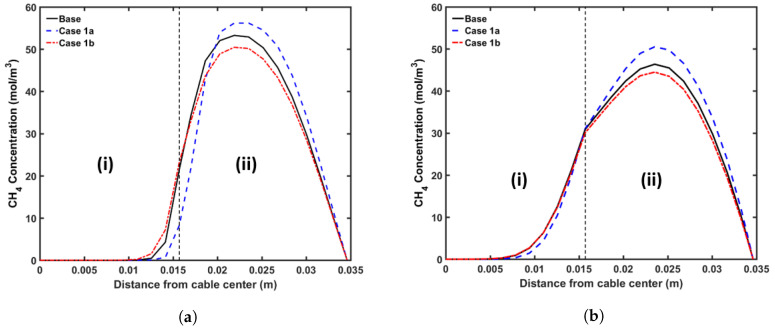
Radial CH_4_ concentration profile for the curing temperature study at (**a**) the curing tube exit, (**b**) the cable take-up point. Segment (i) corresponds to the conductor layer and segment (ii) corresponds to the cross-linked polyethylene (XLPE) and semiconductor (SC) layers.

The following cases were considered for this analysis: (a) Case 1a with a constant curing tube temperature of 350 °C; (b) Case 1b with a constant curing tube temperature of 250 °C. Because the rate of the cross-linking reaction increases exponentially with temperature, using a higher curing temperature allows cable manufacturers to use a higher line speed for cable production. In this analysis, for each case we chose the maximum line speed that yields a cross-linking completion above 99.9% throughout the insulation layer. Using this definition, we used line speeds of 2.4 m·min${}^{-1}$ and 1.2 m·min${}^{-1}$ for Case 1a and Case 1b, respectively. We also used the cooling conditions similar to the base case in this analysis. Figure 11 shows the axial CH${}_{4}$ concentration profile and Figure 12 shows the radial CH${}_{4}$ profile at the curing tube exit and cable take-up point. Table 6 lists the amount of CH${}_{4}$ present in the cable at the cable take-up point.

The results show that the curing temperature had a considerable effect on CH${}_{4}$ generation within the cable. We observed that more CH${}_{4}$ was generated at a higher curing temperature, which is consistent with the reaction selectivity trend shown in Figure 4. The combined effect of the curing temperature and line speed also affected the CH${}_{4}$ distribution. The high curing tube temperature promoted CH${}_{4}$ diffusion because the CH${}_{4}$ diffusion coefficient increased exponentially with temperature, while the cable residence time in each section of the CV line was inversely proportional to the cable line speed. In Case 1a, even though the high curing tube temperature facilitated CH${}_{4}$ diffusion, the 50% reduction of cable residence time compared to the base case limited the amount of CH${}_{4}$ diffused either out of the cable or into the conductor. This resulted in the lower %CH${}_{4}$ removed from the cable and the lower %CH${}_{4}$ in the conductor in Case 1a compared to the base case. In Case 1b, even though the low curing temperature was used, the 25% increase of the cable residence time compared to the base case caused the amount of %CH${}_{4}$ in the conductor to be higher while approximately similar amount of %CH${}_{4}$ was removed from the cable compared to the base case.

#### 5.3.2. Production Line Speed

**Figure 13:**
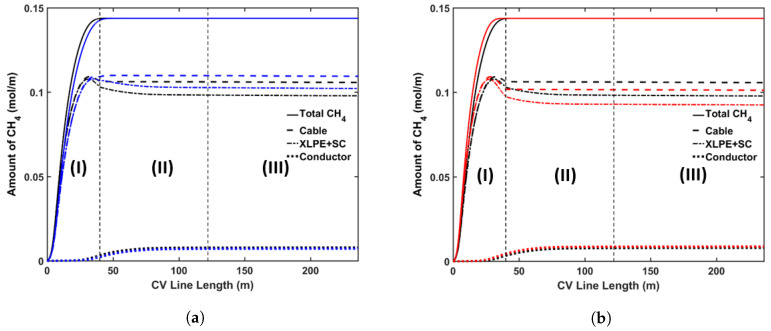
Axial CH_4_ concentration profile across the CV line for the line speed study. The black line represents the base case, the blue line in (**a**) represents Case 2a, and the red line in (**b**) represents Case 2b. Segments (I), (II) and (III) correspond to the curing tube and transition zone, water-cooling, and air-cooling segments, respectively.

**Figure 14:**
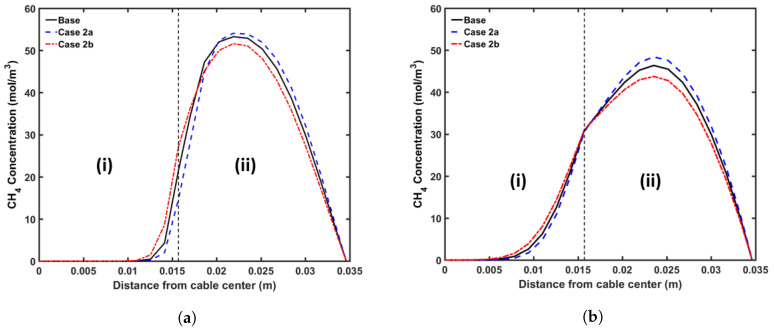
Radial CH_4_ concentration profiles for the line speed study at (**a**) the curing tube exit, (**b**) the cable take-up point. Segment (i) corresponds to the conductor layer and segment (ii) corresponds to the XLPE and SC layers.

We considered the following cases for this analysis: (a) Case 2a with a line speed of 1.8 m·min${}^{-1}$; (b) Case 2b with line speed of 1.4 m·min${}^{-1}$. We applied the curing tube and cooling conditions from the base case in this analysis. Figure 13 shows the axial CH${}_{4}$ concentration profile and Figure 14 shows the radial CH${}_{4}$ profile at the curing tube exit and cable take-up point. Table 7 presents the amount of CH${}_{4}$ existing in the cable at the cable take-up point. We observed cross-linking completion above 99% throughout the insulation layer at the cable take-up point in all studied cases.

The different cable line speed changed the cable residence time in each section of the CV line. This directly influenced the cable temperature and consequently the cross-linking reaction and the diffusion process. The results showed that varying the cable line speed by ±0.2 m·min${}^{-1}$ had a negligible effect on the amount of CH${}_{4}$ generated. On the other hand, we observed noticeable changes in terms of CH${}_{4}$ distribution within the cable. In Case 2a, where the cable line speed was increased by 0.2 m·min${}^{-1}$ relative to the base case, the cable had lower residence time compared to the base case. As the cable heating period within the curing tube decreased, the maximum cable temperature in Case 2a became lower than the maximum cable temperature in the base case. The combination of the temperature difference and the effect of the lower residence time resulted in a reduction in the amount of CH${}_{4}$ removed from the cable or diffused into the conductor. The opposite was true in Case 2b where the line speed was reduced by 0.2 m·min${}^{-1}$ compared to the base case.

#### 5.3.3. Cooling Water Flow Rate

**Figure 15:**
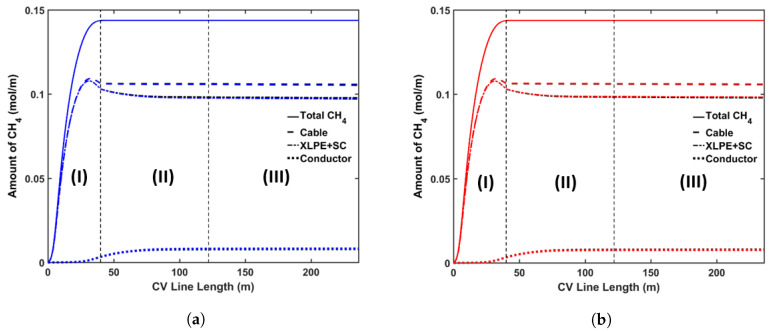
Axial CH_4_ concentration profiles across CV line for cooling-water flow rate study. The black line represents the base case, the blue line in (**a**) represents Case 3a, and the red line in (**b**) represents Case 3b. Segments (I), (II) and (III) correspond to the curing tube and transition zone, water-cooling, and air-cooling segments, respectively.

**Figure 16:**
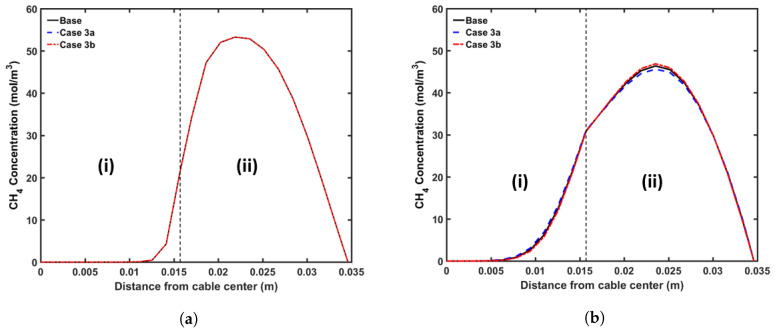
Radial CH_4_ concentration profile for cooling-water flow rate study at (**a**) curing tube exit, (**b**) cable take-up point. Segment (i) corresponds to conductor layer and segment (ii) corresponds to the XLPE and SC layers.

We considered the following cases for the analysis of the water flow rate in the cooling segment: (a) Case 3a with cooling water flow rate of 1 m${}^{3}\cdot$h${}^{-1}$; (b) Case 3b with cooling water flow rate of 10 m${}^{3}\cdot$h${}^{-1}$. The curing tube temperature and cable line speed from the base case were again used in this analysis. Figure 15 shows the axial CH${}_{4}$ concentration profile and Figure 16 shows the radial CH${}_{4}$ profile at the curing tube exit and the cable take-up point. Table 8 lists the amount of CH${}_{4}$ present in the cable at the cable take-up point.

The higher cooling rate resulted in a faster decrease in the cable temperature in the water-cooling section. As the CH${}_{4}$ diffusion coefficient increased exponentially with temperature, using a lower cooling water flow rate increased the amount of CH${}_{4}$ removed from the cable or diffused into the conductor. However, the simulation results demonstrated that these changes were negligible. The cooling rate did not affect the amount of CH${}_{4}$ generated either, as the cross-linking reaction mostly occurred in the curing tube and transition zone.

## 6. Conclusions

In this work, we used a computational diffusion model to estimate CH${}_{4}$ concentrations and their changes during cable production. The model considers various cable heating and cooling mechanisms and heating sources together along the CV line to precisely calculate the temperature changes within the cable due to curing and cooling processes. We then calculated the CH${}_{4}$ generation and transport processes based on the temperature profile and compared them with different production conditions. We have shown that different cable production conditions can affect the generation of byproducts in the PE cable insulation and the transport of byproducts through the cable components. The key findings of our observations are summarized below.

- The amount of CH${}_{4}$ generated during the cross-linking reaction depends highly on the curing temperature because the β-scission reaction becomes more likely than the hydrogen abstraction reaction at higher curing temperatures.
- A relatively large amount of CH${}_{4}$ diffuses out of the cable in the curing tube and transition zone sections, although CH${}_{4}$ released in the cooling section is almost negligible. In the base case considered, approximately 26.5% of the total CH${}_{4}$ is removed from the cable during cable production.
- Some of the CH${}_{4}$ is transferred into the conductor during production. In the base case, approximately 5.5% of the total CH${}_{4}$ flows into the conductor during cable production, and the quantity did not vary significantly when the applied production conditions are varied.
- Due to initial methane release during the curing phase, we consistently observed the non-uniform radial CH${}_{4}$ concentration profile at the cable take-up point, which reasonably resembles the concentration profile obtained from experiments.
- The curing temperature also has a significant effect on CH${}_{4}$ transport because the diffusion coefficient increases exponentially with greater temperature.
- Under similar curing and cooling conditions, varying the production line speed also influences CH${}_{4}$ transport. At lower line speeds, a larger amount of CH${}_{4}$ is eliminated from the cable and is diffused into the conductor.
- The water flow rate in the cooling section has a minor effect on CH${}_{4}$ generation and transport during cable production.

This research presents how the computational model is used to analyze the complex byproduct generation and transport processes within polymer insulation of cables under various heating and cooling processes during the cable extraction and manufacturing. The parametric study results under various conditions can enhance the cable manufacturing procedures regarding the degassing efficiency.

## Figures and Tables

**Table 1:** Continuous vulcanization (CV) line configuration.

Section	Curing Tube	Transition Zone	Water Cooling (1st Section)	Air Cooling
Length (m)	36	4	82	115

**Table 2:** CV line operating condition.

Parameter	Cable Inlet Temp.	Curing Temp.	Line Speed	Water Flow Rate	Water Inlet Temp.
Operating condition	130 °C	300 °C	1.6 m·min${}^{-1}$	3 m${}^{3}\cdot$h${}^{-1}$	25 °C

**Table 3:** Computed kinetic parameters for hydrogen abstraction reaction.

Data Source	$E_{a,a}$ (kJ·mol${}^{-1}$)	$A_{a}$ (L·mol${}^{-1}\cdot$s${}^{-1}$)
Rado [28]	1.8	$1.35\times 10^{7}$
Garrett [29]	15.9	$4.01\times 10^{8}$

**Table 4:** Cable thermal and mechanical properties [3,18,20].

Property	Copper	XLPE	Semiconductor (SC)
Density, ρ (kg·m${}^{-3}$)	8960	922	1050
Specific heat capacity, $C_{p}$ (J·kg${}^{-1}\cdot$K${}^{-1}$)	401	2700	1950
Thermal conductivity, *k* (W·m${}^{-1}\cdot$K${}^{-1}$)	385	0.335	0.53

**Table 5:** Amount of CH${}_{4}$ (mol/m) present in each cable component at the cable take-up point.

Data Source	Conductor	XLPE + SC	Entire Cable	Total Methane
Rado [28]	0.0055 (5.3%)	0.0705 (67.4%)	0.0760 (72.7%)	0.1046 (100%)
Garret [29]	0.0079 (5.5%)	0.0978 (68.0%)	0.1057 (73.5%)	0.1438 (100%)

**Table 6:** Amount of CH${}_{4}$ (mol/m) present in each cable component at the cable take-up point.

Case Code	Conductor	XLPE + SC	Entire Cable	Total Methane
Base	0.0079 (5.5%)	0.0978 (68.0%)	0.1057 (73.5%)	0.1438 (100%)
1a	0.0071 (4.8%)	0.1069 (71.9%)	0.1140 (76.7%)	0.1486 (100%)
1b	0.0078 (5.7%)	0.0937 (67.9%)	0.1014 (73.5%)	0.1379 (100%)

**Table 7:** Amount of CH${}_{4}$ (mol/m) present in each cable component at the cable take-up point.

Case Code	Conductor	XLPE + SC	Entire Cable	Total Methane
Base	0.0079 (5.5%)	0.0978 (68.0%)	0.1057 (73.5%)	0.1438 (100%)
2a	0.0072 (5.0%)	0.1022 (71.1%)	0.1094 (76.1%)	0.1438 (100%)
2b	0.0087 (6.1%)	0.0926 (64.4%)	0.1013 (70.4%)	0.1438 (100%)

**Table 8:** Amount of CH${}_{4}$ (mol/m) present in each cable component at the cable take-up point.

Case Code	Conductor	XLPE + SC	Entire Cable	Total Methane
Base	0.0079 (5.5%)	0.0978 (68.0%)	0.1057 (73.5%)	0.1438 (100%)
3a	0.0082 (5.7%)	0.0971 (67.5%)	0.1053 (73.2%)	0.1438 (100%)
3b	0.0077 (5.4%)	0.0983 (68.4%)	0.1060 (73.7%)	0.1438 (100%)

## Abbreviations

The following abbreviations are used in this manuscript:

AP and CA	Acetophenon and cumyalcohol
CH${}_{4}$	Methane
CV	Continuous vulcanization
DCP	Dicumyl peroxide
HV and EHV	High voltage and extra high voltage
PE	Polyethylene
SC	Semiconductor
XLPE	Cross-linked polyethylene
